# The rumen eukaryotome is a source of novel antimicrobial peptides with therapeutic potential

**DOI:** 10.1186/s12866-021-02172-8

**Published:** 2021-04-08

**Authors:** Lucy A. Onime, Linda B. Oyama, Benjamin J. Thomas, Jurnorain Gani, Peter Alexander, Kate E. Waddams, Alan Cookson, Narcis Fernandez-Fuentes, Christopher J. Creevey, Sharon A. Huws

**Affiliations:** 1 Institute of Biological, Environmental and Rural Sciences, Aberystwyth University, Aberystwyth, Wales SY23 3DA UK; 2 Institute for Global Food Security, School of Biological Sciences, Queen’s University Belfast, 19 Chlorine Gardens, Belfast, Northern Ireland BT9 5DL UK; 3 Institute of Infection and Immunity, St. George’s University of London, Cranmer Terrace, London, SW17 0RE UK

**Keywords:** Resistance, Antimicrobials, Antimicrobial peptide, Rumen, Microbiome, Eukaryotes, Eukaryotome

## Abstract

**Background:**

The rise of microbial antibiotic resistance is a leading threat to the health of the human population. As such, finding new approaches to tackle these microbes, including development of novel antibiotics is vital.

**Results:**

In this study, we mined a rumen eukaryotic metatranscriptomic library for novel Antimicrobial peptides (AMPs) using computational approaches and thereafter characterised the therapeutic potential of the AMPs. We identified a total of 208 potentially novel AMPs from the ruminal eukaryotome, and characterised one of those, namely Lubelisin. Lubelisin (GIVAWFWRLAR) is an α-helical peptide, 11 amino acid long with theoretical molecular weight of 1373.76 D. In the presence of Lubelisin, strains of methicillin-resistant *Staphylococcus aureus* (MRSA) USA300 and EMRSA-15 were killed within 30 min of exposure with ≥10^3^ and 10^4^ CFU/mL reduction in viable cells respectively. Cytotoxicity of Lubelisin against both human and sheep erythrocytes was low resulting in a therapeutic index of 0.43. Membrane permeabilisation assays using propidium iodide alongside transmission electron microscopy revealed that cytoplasmic membrane damage may contribute to the antimicrobial activities of Lubelisin.

**Conclusions:**

We demonstrate that the rumen eukaryotome is a viable source for the discovery of antimicrobial molecules for the treatment of bacterial infections and further development of these may provide part of the potential solution to the ongoing problem of antimicrobial resistance. The role of these AMPs in the ecological warfare within the rumen is also currently unknown.

**Supplementary Information:**

The online version contains supplementary material available at 10.1186/s12866-021-02172-8.

## Background

Increasing bacterial resistance to existing antimicrobials has led to a global human health crisis, which requires exploration of alternatives to existing antibiotics [1–3]. The O’Neill report stated that by 2050, the burden of deaths from antimicrobial resistance (AMR) will be approximately 10 million lives each year [4]. The report also noted that alternative strategies, such as phage therapy, lysins, antibodies, and antimicrobial peptides (AMPs) should be developed to combat the challenge of multi-drug resistant bacteria [4].

AMPs are a distinct set of innate low molecular weight molecules that are found in living organisms and have broad spectrum activity against various bacterial species, eukaryotes (fungi, protozoa) and enveloped viruses [5]. They act rapidly, curtailing early onset of resistance. AMPs are classified according to their amino acid components and structure, which differ extensively. They are, however, easy to identify because they share some key characteristics, such as multiple cationic charges and high hydrophobicity [6, 7]. Most AMPs engage with the bacterial bilayer membrane, which means they can also be effective against inactive viable bacteria, giving them an advantage over classic antibiotics that require prolonged treatment to be effective against dormant bacteria [8]. In eukaryotic cells, AMPs have been described as efficacious molecules in the first line of chemical defence against other microbial organisms [5, 9–11].

Although antimicrobials have been identified in some eukaryotes [12–14], there is a need to intensify efforts due to the increase in antimicrobial drug resistance. Microbiomes possess an array of microorganisms offering a unique resource for antimicrobial discovery. The rumen is an example of a complex microbiome made up of eukaryotic microorganisms (anaerobic fungi and protozoa), bacteria, viruses and methanogenic archaea, all of which have intricate interrelationships. The rumen microbiome has been shown to be a source of antimicrobials. Bacteriocins and bacteriocin-producing strains have been isolated from ruminal bacteria [15, 16]. More recently AMPs, Buwchitin, Lynronnes-1, 2 and 3, HG2 and HG4, coupled with non-ribosomal peptides were identified from rumen bacterial metagenome libraries or sequences [17–19]. The rumen derived antimicrobials and AMPs identified so far have been mostly from prokaryotes, while the rumen eukaryotome remains largely underexplored. In this study, we hypothesised that rumen eukaryotes possess numerous AMPs, enabling them to be able to compete in the rumen. We identified and characterised novel AMPs from metatranscriptome sequencing data obtained from the rumen eukaryotome. To date, there are no published AMPs of rumen eukaryotic origin to our knowledge.

## Results

### In silico mining of antimicrobial peptides and screening against bacterial strains

Using bioinformatic analytic tools and online databases, 208 potential AMP candidates were identified from a rumen eukaryotic metatranscriptomic sequence dataset (Supplementary Table 1). These sequences formed the basis of our AMP library to be screened. For the initial high-throughput antimicrobial activity screening, peptides were synthesised using the spot technique as it allows for synthesis and screening of a high number of peptides at an affordable cost. Peptide activity was investigated against *Salmonella enterica* serovar Typhimurium SL1344, *Escherichia coli* K12, epidemic methicillin-resistant *Staphylococcus aureus*-15 (EMRSA-15) *and Pseudomonas aeruginosa* strain H1001 (containing the lux operon resulting in bioluminescence when the bacteria are viable). Thirteen of these 208 peptides showed promising activity in the spot screen, one of which - Lubelisin (GIVAWFWRLAR) was selected for further testing. The coding sequence(s) (CDS) from the mRNA transcripts in the dataset from which Lubelisin (three transcripts) was derived are available in the GenBank database under accession numbers MK286889, MK286890 and MK286891. A homology search in the Antimicrobial peptide database APD3 [20] reveal that Lubelisin has 50% similarity to peptide P15a identified from rumen bacteria [18], 44% similarity to CcAMP1 (from the arthropod *Coridius chinensis*), 42.85% to buCATHL4B (cathelicidin from *Bubalus bubalis*), 41.66% to Peptide 536_2 (predicted from medicinal leech *Hirudo medicinalis*) and 38.46% to Tet037 (a synthetic peptide-). Sequence alignment of Lubelisin and these top five homologous AMP hits on the APD3 database using Clustal [21] is included in Supplementary Table 1. The sequences of transcripts of the parent protein from which Lubelisin was derived were compared against the MiBIG database [22] using multiple sequence alignment (MUSCLE) [23] and sequence similarity clustering (CD-HIT) [24] at 80 and 90% similarity thresholds and no hits to known biosynthetic gene clusters could be resolved. The length of the Lubelisin transcripts (682, 688 and 724 bp) restricted analysis using other tools such as antiSMASH [25] which require a minimum sequence length of 1000 bp.

### Determination of minimum inhibitory concentration (MIC) and minimal bactericidal concentration (MBC)

The MICs of these 13 AMPs against a range of bacterial pathogens were determined. Based on this MIC data (Table 1), one AMP, namely Lubelisin, was chosen for further characterisation due to it's low MIC values. Lubelisin exhibited varying degrees of antibacterial activities against the microorganisms tested. It was particularly more active against Gram-positive bacteria strains with MIC range between 8 to 64 μg/mL (Table 1). Lubelisin had an MIC of 8 μg/mL and MBC of 32 μg/mL respectively against *S. aureus* strains. It was also active against *E. coli* K12 (MIC of 64 μg/mL, MBC 128 μg/mL) and a sensitive hospital isolate of *Acinetobacter baumannii* (MIC*,* 32 μg/mL, MBC 64 μg/mL). A higher MIC of 128 μg/mL was observed for drug resistant *A. baumannii* isolates. Lubelisin was least active against *Pseudomonas aeruginosa* strains PAO1 and H174 with MICs of 128 μg/mL and 256 μg/mL respectively.

**Table 1:** Minimum Inhibitory Concentrations of the idenfies Antimicrobial Peptides against a range of bacterial pathogens (μg/mL) (highest concentration tested = 512 μg/mL) ‘-’ not tested. *OXA* Oxacillin, *IMI* (imipenem), *MER* (meropenem)

Peptide ID	***S. aureus*** MSSA	***S. aureus*** USA300	***S. aureus*** ATCC 33591	***S. aureus*** EMRSA-15	***Ent. faecalis***	***B. cereus***	***E. coli K12***	***Sal. Tyhimurium***	***P. aeruginosa*** PAO1	***P. aeruginosa*** H174	***A. baumannii*** DSMZ 30007	***A. baumannii*** S27379 Sen	***A. baumannii*** S15785 OXA-23/0XA-50	***A. baumannii*** IMI/MER-R
Lub_323	512	> 512	> 512	> 512	512	> 512	512	512	512	512	512	512	512	512
Lub_353	256	256	256	> 512	256	512	128	256	256	512	256	256	512	128
Lub_364	> 512	> 512	> 512	> 512	256	> 512	256	> 512	> 512	> 512	256	256	512	256
Lub_376	> 512	> 512	> 512	> 512	> 512	> 512	> 512	> 512	> 512	> 512	> 512	> 512	> 512	> 512
Lubelisin (Lub_383)	8	8	8	8	16	64	64	128	128	256	128	32	128	128
Lub_405	> 512	> 512	> 512	> 512	> 512	> 512	> 512	> 512	> 512	> 512	> 512	> 512	> 512	> 512
Lub_424	> 512	> 512	> 512	> 512	512	> 512	256	512	> 512	512	> 512	> 512	512	> 512
Lub_443	512	256	> 512	256	256	512	256	> 512	> 512	> 512	512	256	256	256
Lub_444	256	256	256	128	128	> 512	256	> 512	> 512	512	512	256	512	512
Lub_454	> 512	> 512	> 512	> 512	> 512	> 512	> 512	> 512	> 512	> 512	> 512	> 512	> 512	> 512
Lub_458	> 512	> 512	> 512	> 512	512	> 512	512	> 512	> 512	> 512	512	512	512	512
Lub_523	512	> 512	> 512	> 512	> 512	> 512	> 512	> 512	> 512	> 512	> 512	512	> 512	512
Lub_526	> 512	> 512	512	> 512	> 512	> 512	> 512	> 512	> 512	> 512	> 512	> 512	> 512	> 512
Vancomycin	2	2	2	2	2	2	–	–	–	–	–	–	–	–
Levofloxacin	–	–	–	–	–	–	0.03	0.12	0.06	0.5	0.25	0.12	> 512	256
Polymyxin B	–	–	–	–	–	–	0.5	1	2	1	1	1	0.5	1

### AMP structure predictions and biophysical parameters

**Fig. 1:**
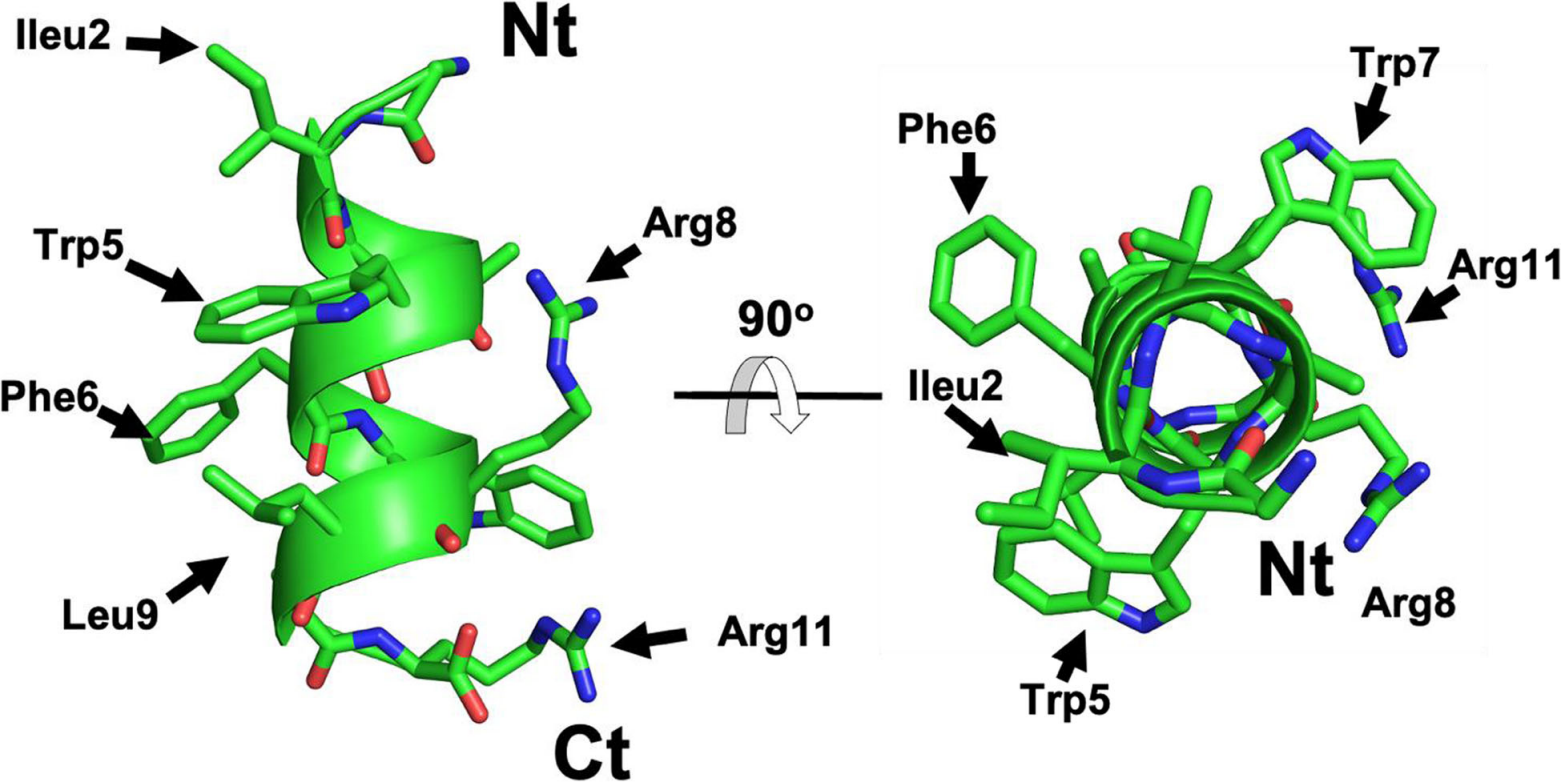
Predicted 3D structure of Lubelisin. Main and side chains shown in ribbon and stick representation and coloured according to atom type: Carbon, Oxygen and Nitrogen in green, red and blue respectively. N- and C termini and some residues also shown in two different orientations rotated by 90 degrees respectively. Figure rendered using PyMol

Lubelisin (GIVAWFWRLAR, molecular weight 1373.76) is 11 amino acids (AAs) in length, is positively charged (+ 2) and highly hydrophobic with 72% hydrophobic residues, features, which are common characteristics of antimicrobial peptides (Fig. 1). Molecular modelling of the peptide 3D structure based on a precomputed library of short structural fragments assembled and sampled using a Monte Carlo simulation revealed that Lubelisin converges into an amphipathic α-helix with a clear pattern of spatial segregation of hydrophobic versus charged residues typical of many known antimicrobial peptides (Fig. 1). Circular dichroism studies in different solutions/environments and other methods, which give the residue-specific information may be required to confirm the accuracy of the predicted structure upon peptide interaction with membranes and how this affects peptide activity.

### Time-kill assays

**Fig. 2:**
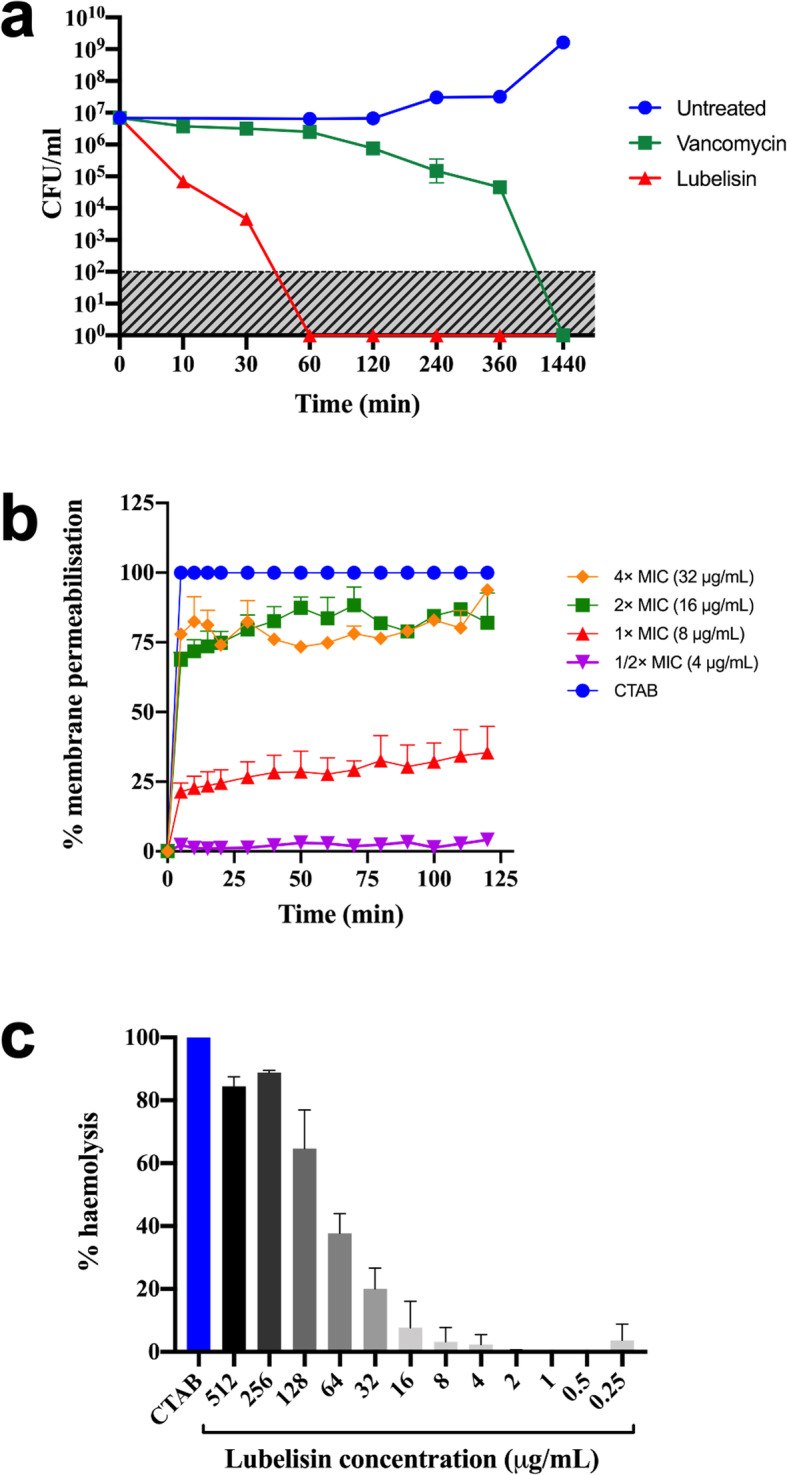
Activity of Lubelisin: time dependent kill at 3× MIC concentration against EMRSA-15 cells (**a**); Membrane permeabilisation activity against EMRSA-15 (**b**); haemolytic activity of Lubelisin against human erythrocytes at a range of concentrations (**c**). For a-c, data are expressed as mean ± standard deviations from 3 (**a**, **c**) and 2 (**b**) independent replicates respectively, and if error bars cannot be seen, this is since they were smaller than the symbols. Shaded area in (**a**) indicates limit of detection

To characterise the bactericidal or bacteriostatic activity of Lubelisin, exponential-phase MRSA cells, EMRSA-15 and USA300 cells were challenged with a 3× MIC concentration of the peptide. Post-challenge viability was assessed by determination of CFU/mL. Lubelisin at 3× MIC was rapidly bactericidal against EMRSA-15 with ≥10^3^ CFU/mL reduction in viable cells (Fig. 2a) and ≥ 10^4^ CFU/mL reduction in MRSA USA300 viable cells (Supplementary Table 1) within 30 min of exposure to the peptide. The comparator antibiotic, vancomycin at 3× MIC produced between ≥10^3^ CFU/mL reduction in the EMRSA-15 cells after 6 h, attributable to differences in kill kinetics and mode of action [26].

### Serial passage for selection of resistance

No resistant mutants were recovered after 26 days of serial passage of EMRAS-15 cells in the presence of sub-MIC levels of Lubelisin (see daily MICs obtained in serial passage assay in Supplementary Table 1).

### Membrane permeabilisation

Membrane permeabilisation ability of Lubelisin in EMRSA-15 cells evaluated using the propidium iodide method revealed that Lubelisin had a concentration dependent membrane permeabilising effect (see membrane permeabilisation for all concentration ranges in Supplementary Table 1). Compared to CTAB, Lubelisin at MIC concentration induced ~ 25% membrane permeabilization in EMRAS-15 cells, and > 75% at 2× and 4× MIC concentrations within a few minutes of exposure (Fig. 2b). This may indicate that Lubelisin employs membrane permeabilisation in its antimicrobial activity against the pathogen.

### Haemolytic activity

The haemolytic activity of Lubelisin against human RBC was assessed as an indication of cytotoxicity towards mammalian cells. Lubelisin exhibited a concentration dependent haemolysis (Fig. 2c) causing little haemolysis at sub-MIC concentration and up to 90% haemolysis at the highest concentration (512 μg/mL) tested when compared to the control agent 0.1% Triton X-100 (Fig. 2c). At MIC concentration (8 μg/mL), Lubelisin caused 4.8% haemolysis to human RBC resulting in a low therapeutic index of 0.43. The haemolytic activity of Lubelisin against sheep erythrocytes was also concentration dependent with ~ 10, 20 and 30% haemolyis at MIC, 2× and 3× MIC concentrations respectively (Supplementary Table 1).

### Transmission electron microscopy

**Fig. 3:**
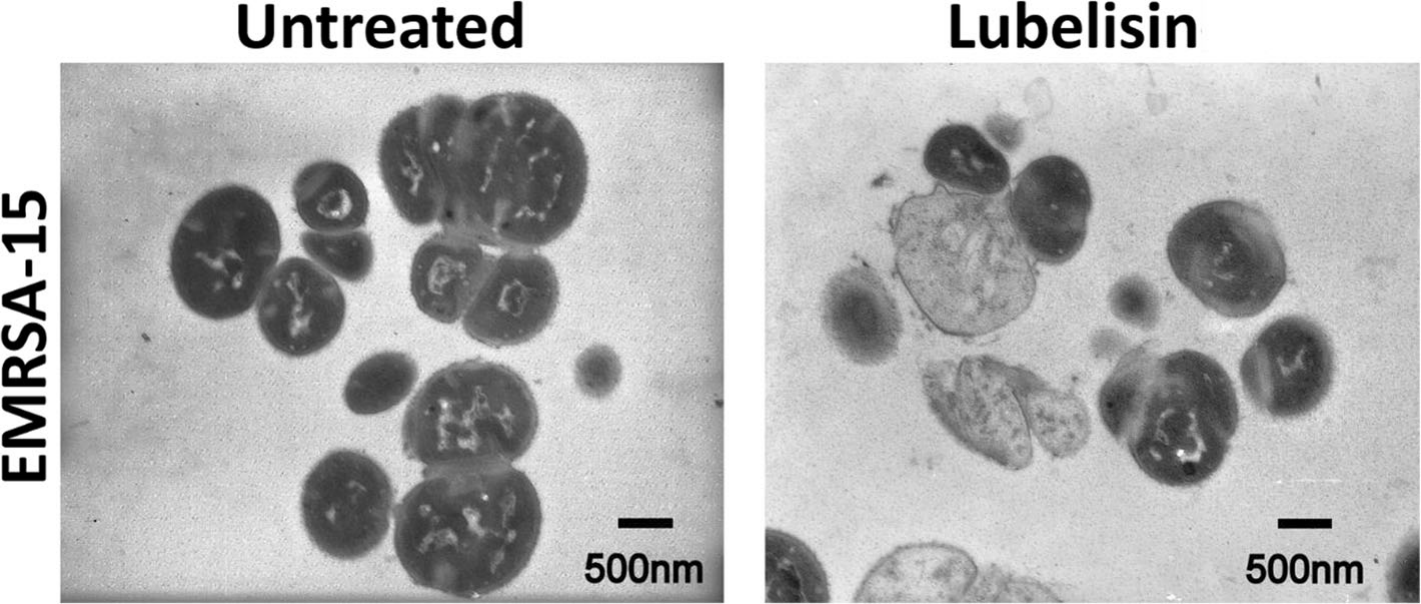
Transmission electron micrographs of Lubelisin against EMRSA-15 cells. Untreated and treated cells after 30 min exposure to Lubelisin at 3× MIC concentration. Scale bars are 500 nm as shown on micrographs

Transmission electron micrographs (useful for visualising the interior sections of single cells) revealed varying morphological changes to EMRSA-15 cells treated with Lubelisin at 3× MIC concentration after 30 min exposure to the AMP (Fig. 3). Compared to the untreated control cells, observed to have intact cytoplasmic membrane structures, changes to cell morphology including ‘emptying’ and/or ‘ghost cells’ effects were evident in cells treated with Lubelisin, indicating potential membrane disruption and subsequent loss of cytoplasmic content (Fig. 3).

## Discussion

Multi-drug-resistant microorganisms are increasing in abundance resulting in high mortality rates and a global human health threat. This challenge of increasing resistance to known antimicrobials can, in part, be circumvented by discovery and development of novel alternatives, including AMPs [4]. In this study, we used computational in silico methods to bio-prospect for AMPs within metatranscriptomic sequencing data obtained from the rumen eukaryotic community. One cationic AMP, Lubelisin with promising antimicrobial activity was selected and further characterised. Cationic AMPs such as Lubelisin with amphipathic structures and high hydrophobicity are known to be membrane-active, with enhanced Gram-positive antibacterial activity and are linked to a reduction of peptide specificity for Gram-negative bacteria [27–29]. Moreover, the positively charged surface of the peptide may interact with bacterial membranes. Tryptophan residues in Lubelisin may play a role in its antimicrobial activities, as they have been observed to confer broad and effective antimicrobial properties and interact with the bilayer interfacial region of bacterial membranes [30, 31]. Natural antimicrobial peptides can present an amphipathic helical conformation although others, such as beta-hairpin, hybrid beta/helical and other extended conformations, are also possible [32]. Helical conformation is among the most common among antimicrobial peptides [33]. Examples of helical antimicrobial peptides include spinigerins [34], magainins [35], moricins [36] and others. Given the regularity of alpha helices, the structural similarity among any given pair of helices will be necessarily very high, particularly on short helices, Sequence similarity of Lubelisin indicated homology to known AMPs including P15a (from rumen bacteria- 50%), CcAMP1 (from the arthropod *Coridius chinensis*- 44%), buCATHL4B (cathelicidin from *Bubalus bubalis*- 42.85%), Peptide 536_2 (predicted from medicinal leech *Hirudo medicinalis*- 41.66%) and Tet037 (a synthetic peptide- 38.46%).

The other 12 AMPs identified in the spot synthesis screen showed poor activity when purified versions were tested for antibacterial activity. This may indicate that using the luminescent *P. aeruginosa* H1001 in a buffer solution is very sensitive to favourable amino acid combinations within 4 h, but compared to short cationic peptides in other studies [18], where the assay showed very good correlation, here as also observed in other studies [37], the results from the spot synthesis screen could not be directly correlated to the antimicrobial activity of most (12 of the 13) AMP candidates identified from the spot screen.

Lubelisin demonstrated MICs between 8 to 64 μg/mL against Gram-positive bacteria strains and higher MICs between 32 to 256 μg/mL against Gram-negative strains. Higher MICs were observed for clinical isolates of multidrug drug resistant *A. baumanni* strains (128 μg/mL) compared to the sensitive isolate (32 μg/mL). The clinical *A. baumannii* isolates exhibiting higher MICs for Lubelisin are carbapenem resistant strains possessing an inherent class D β-lactamase gene (blaOXA-51-like) as well as the distantly related blaOXA-23 genes [38]. Although colistin is the last resort for the treatment of infections with carbapenem-resistant (CR) Gram-negative bacteria including *P. aeruginosa* and particularly *A. baumannii* (CRAB), strains resistant to colistin have been isolated globally [39–41]. It has been shown that lipopolysaccharide-deficient Gram-negative bacteria including clinical isolates of *A. baumannii* can rapidly develop resistance to polymyxin antibiotics by complete loss of the initial binding target, the lipid A component of lipopolysaccharide (LPS- considered to be essential for the viability of Gram-negative bacteria) [40] following mutation within one of the first three genes of the lipid A biosynthesis pathway: *lpxA*, *lpxC*, and *lpxD* [42]. Modification of lipopolysaccharide (LPS) by upregulation of the *pmrCAB* operon results in the synthesis and addition of positively charged phosphoethanolamine to the LPS. An increase in positive charge of the LPS leads to a decrease in the binding between colistin (positive charge) and lipid A (negative charge) of the LPS, thus resulting in colistin resistance [42]. This modification has been observed in all colistin-resistant *A. baumannii* isolates, but none of the corresponding colistin-susceptible isolates [43–45]. Like colistin, Lubelisin is positively charged and membrane acting and so the poor MICs observed for Lubelisin in these strains compared to the sensitive strain may be indicative of a modification in their LPS. This higher MIC in Gram-negative MDR strains has also been observed in other rumen microbiome derived AMPs [18].

Lubelisin demonstrated rapid antimicrobial activity causing between ≥10^3^ and ≥ 10^4^ CFU/mL reduction in viable cells within 30 min against the Gram-positive bacteria, EMRSA-15 and MRSA USA300 respectively. This rapid action induced by Lubelisin is common with many cationic AMPs including colistin, avian β-defensins and Lynronne-1 [18, 46] and it is thought that this reduces the likelihood of resistance developing against the AMPs although resistance to colistin in Gram-negative bacteria has been widely reported [40]. Serial passage of EMRSA-15 cells in the presence of sub-MIC levels of Lubelisin failed to produce resistant mutants (MIC values remaining within one to two-fold shifts), suggesting a non-specific mode of action [47]. Lubelisin had a low therapeutic index of 0.43 an indication that it may have low bacterial specificity. Modifications to improve the therapeutic index of Lubelisin will be needed to improve its usefulness for the treatment of non-topical infections. Well researched integrated approaches for developing peptide analogues with desired characteristics (structurally simple, broad spectrum antibacterial activity and non-cytotoxic) [46] will be required to improve the therapeutic application of Lubelisin.

Membrane permeabilisation of ~ 75% in EMRA-15 cells was observed when exposed to Lubelisin compared to CTAB. A time-dependent increase in permeabilization was observed from 0 to 120, however, this did not increase significantly after 30 min, indicating a fast-action mode of Lubelisin. Several morphological changes including disintegration of the cytoplasmic membrane and emptying of cellular content were observed in TEM micrographs of Lubelisin treated EMRA-15 cells. This may indicate that loss of viability in Lubelisin treated EMRA-15 cells, is induced by cytoplasmic membrane damage among other factors, causing interference with the cell division mechanism of the bacteria and targeting intracellular structures. Many studies have indicated that amphipathicity of alpha-helical peptides (in which hydrophobic residues interact with membrane lipid components while hydrophilic regions either bind with the phospholipid head groups or form the lumen of a membrane pore) is a key characteristic required for membrane permeabilization and consequently antimicrobial activity [10]. The importance of these peptides in the ecological interactions within the rumen remain unknown.

## Conclusions

The antimicrobial peptide Lubelisin characterised in this study is novel and efficacious in in vitro experiments against clinically relevant human pathogens, particularly methicillin-resistant *S. aureus* strains, rendering them as potential leads and/or templates for development of alternative treatment strategies for infections caused by these pathogens. The identification of Lubelisin also supports the hypothesis that rumen eukaryotes possess AMPs and are a viable alternative resource for the discovery of novel antimicrobials relevant in the fight against multi-drug resistant bacteria.

## Methods

### Sample preparation and RNA extraction

We obtained metatranscriptomic data for the rumen eukaryotes from an experimental design previously reported, whereby we analysed diversity (metataxonomy) and function (metatranscriptomics) from fresh perennial ryegrass attached bacteria (NCBI bioproject ID PRJNA274256) [48]. Briefly, fresh perennial ryegrass was incubated in the rumen of three cannulated, non-lactating Holstein x Friesian cows, using the nylon bag method and under home office licence. Two bags were removed at 1, 2, 4, and 6 h post incubation, and residual forage was washed and stored at − 80 °C. In order to obtain metatranscriptome data for the polyA eukaryotic fraction, rumen samples from the experiment were frozen and ground to a fine powder under liquid nitrogen before RNA was extracted using a hot phenol method [49]. Essentially, aquaphenol (10 mL) was added to the ground sample prior to incubation at 65 °C for 1 h. Tubes were inverted before chloroform was added (5 mL). Tubes were centrifuged (5000×*g*, 30 min, 20 °C) before upper phase was removed then the procedure was repeated by addition of more chloroform (5 mL) and centrifugation as described. Lithium chloride (2 M final concentration) was then added, to remove any contaminating DNA, and samples stored overnight at 4 °C. Samples were subsequently centrifuged (13,000×*g*, 30 min, 4 °C) and supernatant discarded, then the procedure was repeated with addition of lithium chloride to ensure all DNA was removed. Once the supernatant was discarded the pellet was resuspended in ice cold 80% ethanol and centrifuged (13,000×*g*, 15 min, 4 °C), this was repeated twice before the pellet was air dried and resuspended in molecular grade water. Absence of DNA in all sample RNA extracts was checked using PCR as described in Huws et al. (2016) [48], using non-barcoded primers and subsequent agarose gel electrophoresis. Quality and quantity of retrieved RNA was checked using the Experion automated electrophoresis system and RNA StdSens chips (Bio-rad, Hemel Hempstead, United Kingdom).

### RNA enrichment

The RNA was enriched for Polyadenylated mRNA (eukaryotic fraction) using a Poly(A) Purist™ MAG kit (Life Technologies, USA) (produced by eukaryote fraction only), following the manufacturer’s guidelines; this was repeated twice to minimise prokaryotic DNA and rRNA carryover. An equal volume of 2X Binding Solution was added to each sample and mixed thoroughly (thus doubling the initial sample volume). A mass of Oligo (dT) MagBeads equivalent to the amount of RNA was added to a clean tube, then captured and pulled out of the storage solution using a magnetic stand (Thermo-Fisher Scientific, USA). The storage buffer was removed and discarded then Wash solution 1 added at a ratio of 500 μL per mg of beads. The tube was removed from the stand and the beads resuspended, the tube was then put back on the stand, the Magbeads captured and solution removed. The RNA in binding solution was then added to the beads, mixed thoroughly and they were then incubated at 75 °C for 5 min. The tubes were then incubated at RT for 1 h on a shaker. Next, the tubes were put back into the magnetic stand to capture the beads and the supernatant removed. The beads with bound RNA were washed with Wash solution 1, mixed thoroughly and then placed back on the magnetic stand. The solution was then discarded, and the process repeated. Using Wash Solution 2 the process was repeated a further two times.

Finally, the Poly(A) RNA was recovered by removing the tubes from the magnetic stand, adding 200 μL of pre-heated RNA storage solution and mixing well. The tubes were then placed back in the magnetic stand to capture the beads, and the RNA in solution was removed into a new tube. A further 200 μL of warm RNA storage solution was added and the process repeated. The Poly(A) RNA was then precipitated using 40 μL 5 M Ammonium Acetate, 1 μL Glycogen and 1.1 mL 100% EtOH overnight at − 20 °C. The RNA was recovered by centrifugation at 12,000 rpm for 30 min and 4 °C. The supernatant was discarded, and the tube centrifuged again briefly to remove all remaining solution. The pellet was washed using 1 mL 70% EtOH: vortexed briefly, centrifuged at 12,000 rpm for 10 min at 4 °C and supernatant discarded. The RNA pellet was then resuspended in 20 μL of the RNA Storage solution and stored at − 80 °C.

Eukaryotic 18S rRNA was further minimised using the RiboMinus Plant Kit (Invitrogen, California, USA), according to manufacturer protocols. Firstly 225 μL of magnetic beads per sample was placed in a 1.5 mL microcentrifuge tube and brought out of solution using a magnetic stand, the storage buffer was discarded, and the beads were washed twice using 225 μL of RNase-free water and 60 μL of magnetic bead resuspension solution. Once the remaining wash buffer had been discarded, 65 μL of magnetic beads were added to a fresh tube. RiboGuard RNase Inhibitor solution (1 μL) was also added per sample. To each tube, the following reagents were added: 4 μL Ribo-Zero Reaction buffer, 20 μL RNA sample, 10 μL Ribo-Zero Removal solution and RNase-free water to a total volume of 40 μL. The samples were then incubated at 68 °C for 10 min and at room temperature for 5 min. Next, the washed magnetic beads were added to the sample, vortexed and incubated at room temperature for 5 min before placing at 50 °C for a further 5 min. The samples were then placed on the magnetic stand and the supernatant containing the depleted RNA moved to a fresh tube.

Finally, 16S rRNA was further minimised using Ribo-Zero rRNA removal kit (bacteria) (Illumina, California, USA) according to manufacturer protocols. The previously purified RNA was added to 10 μL of RiboMinus™ Probe (15 pmol/μL) and 100 μL of Hybridization buffer and incubated at 75 °C for 5 min. The sample was then cooled in a 37 °C water bath over 30 min. The RiboMinus™ Magnetic beads (750 μL) were placed into a 1.5 mL microcentrifuge tube and set in the magnetic rack and the storage buffer aspirated and discarded. Next, the beads were washed twice using 750 μL DEPC- H_2_O before resuspension in 750 μL of Hybridization buffer. Beads (250 μL) were then transferred into a new tube and incubated at 37 °C whilst the remainder (500 μL) were placed in the magnetic stand. The supernatant was removed and discarded and then the beads resuspended in 200 μL of hybridization buffer and incubated at 37 °C. Next, the sample was added to 500 μL of beads, mixed and incubated at 37 °C for 15 min, after which the tubes were placed onto the magnetic stand and the supernatant moved to a fresh tube. The second aliquot of prepared magnetic beads (250 μL) was set on the magnetic rack, supernatant removed and discarded before adding the beads to the previous supernatant containing the purified RNA. After mixing and incubation at 37 °C for 15 min, the tube was placed onto the magnetic stand and the supernatant moved to a fresh tube. An ethanol (EtOH) precipitation was then used to purify the RNA. Two volumes of cold EtOH and 2 M ammonium acetate were added, the solution mixed and placed at − 20 °C overnight. The RNA was then recovered by centrifugation at 4 °C for 30 min at 13,000 rpm. The supernatant was then gently removed and a further 1 volume of cold 70% EtOH added. The solution was then centrifuged again at 4 °C for 10 min at 13,000 rpm and the process repeated. The RNA was centrifuged once more at 4 °C for 5 min at 13,000 rpm and any remaining supernatant discarded. The pellet was then briefly air dried and resuspended in DEPC-H_2_O. The purified products were then checked for purity and quantity using the Experion automated electrophoresis system (Bio-Rad Laboratories, UK).

### Sequencing

The mRNA was prepared for sequencing using a TruSeq stranded mRNA library prep kit (Illumina, California, USA) following manufacturer guidelines. Library sequencing was completed using the Illumina HiSeq 2500 (Illumina, California, USA) 100 bp paired-end sequencing. Sequencing data was quality checked using FastQC (Version 0.69; Babraham Bioinformatics, UK) and then trimmed using a sliding window trim with Trimmomatic (version 0.36.0) [50, 51]. Sliding window size was set at 4 bps and average quality required was set at 20. Assembly of sequence data was carried out using Trinity (version 0.0.1) [52] at default parameters. A metatranscriptomic approach was utilised to obtain rumen eukaryotic sequences due to the increased difficulties of binning rumen eukaryotic sequences with confidence from shotgun metagenomic sequences. Sequences are deposited in the Short Read Archive under Bioproject: PRJNA563675; Biosample: SAMN12684929: HAN4BADXX.

### In silico mining and prediction of antimicrobial peptides

Putative AMP candidates were identified from the quality checked and assembled metatranscriptomic data using publicly available analysis tools including the Antimicrobial Peptide Database (APD3) [20], the Antimicrobial Sequence Scanning System (AMPA) [53], BACTIBASE [54] and the Collection of Anti-Microbial Peptides modelling tools (CAMPR3) [55] to identify sequences of interest and bioactive regions suitable for further characterisation. A total of 208 potential AMPs were predicted, which were then chemically spot synthesized at ≥60% purity and screened for antimicrobial activity as described below [56].

### AMP structure predictions

Molecular modelling of the 3D structures of peptides showing potential therapeutic potential was carried out using the PEP-FOLD structural prediction method [57]. The best models for each peptide were selected based on the OPEP force field [58] and the PEP-FOLD score. Predicted peptide structures were visualized using the PyMOL v1.7.6 program [59]. Hydrophobicity (H) and Hydrophobic moment (μH) were calculated by using the program heliquest [60], as window size the whole length of peptide was used in case of the 11 and 12mer peptides, window size 12 for the 13mer peptides, where the first window was reported.

### Antibacterial activity screening of spot-synthesised AMPs

The dissolved (in sterile distilled water) spot synthesised peptides in 96 well plates (Greiner Bio One Ltd., Stonehouse, UK), were screened against diluted overnight cultures (final concentration at 5 × 10^5^ Colony Forming Units per millilitre (CFU/mL) of luminescent *P. aeruginosa* H1001 as previously described [61]. Activity against *P. aeruginosa* H1001 strain was assessed by luminescence reading (excitation/emission spectra of 470/695 nm) using the Hidex Sense Plate Reader Software 0.5 pre-release (LabLogic, Sheffield UK). Activity against non-luminescent strains (epidemic methicillin-resistant *S. aureus* EMRSA-15, *Salmonella Typhimurium* and *Escherichia coli* K12) was assessed by fluorescence readings (excitation/emission spectra of 560/590 nm) after the addition of resazurin dye (100 μM final concentration). Peptides that caused ≥75% reduction in fluorescence or luminescence relative to the growth controls were selected as having antimicrobial activity. The Peptides Extension Package of the MATLAB toolbox [62]. SciXMiner was used for the computational analysis [63].

### Peptide synthesis

Pure peptide synthesis (> 95% purity) on resin was completed for peptides showing potential antibacterial activity from crude spot synthesis via Genscript Inc. USA, using 9-fluorenylmethoxycarbonyl for solid phase peptide synthesis. Quality analyses of the peptides were validated using high-performance liquid chromatography and mass spectrometry (see Supplementary Table 1). All pure AMPs were dissolved in sterile distilled water. The antimicrobial activity of pure AMPs were ascertained followed by cytotoxicity, resistance and mechanistic studies as described below.

### Determination of minimum inhibitory concentration (MIC) and minimal bactericidal concentration (MBC)

MICs against a range of clinically important Gram-positive and Gram-negative bacteria strains including methicillin-sensitive *Staphylococcus aureus* (MSSA) RN4220 epidemic methicillin-resistant *S. aureus*-15 (EMRSA-15), methicillin-resistant *S. aureus* USA300 (MRSA USA300), *Bacillus cereus*, *Enterococcus faecalis* JH2–2, *Escherichia coli* K12, *Salmonella enterica* serovar Typhimurium SL1344, *P. aeruginosa* PAO1, and hospital isolates of *Acinetobacter baummannii* were determined in quadruplicate by a modified microdilution broth method [64] with a final bacterial inoculum concentration of 5 × 10^5^ CFU/mL utilised as the test organisms. Cation-adjusted Mueller Hinton (MH) broth was innoclated overnight with a single colony of the tested microorganisms, subsequently grown for 16–24 h with shaking at 225 rpm, and further diluted in the same broth to a final concentration of 5  ×  10^5^ CFU/mL. The bacterial suspension was added to sterile U-bottomed 96-well plates, and the peptides and control antibiotics to be tested were dissolved in sterile distilled water and serially diluted in the wells. The MICs were recorded as the lowest peptide concentration that inhibited growth after incubation for 18 h [65]. Minimal bactericidal concentration (MBC, defined as the lowest concentration of compound that kills > 99.9% of bacteria cells) assessed by colony forming units was determined by plating serially diluted cultures from wells from the MIC plates.

### Time-kill assays

Time-dependent kill assays were adapted from standard procedures [66] and were all performed as previously using exponential-phase cultures of EMRA-15 MRSA and USA300 grown in MHB (1 × 10^6^ CFU/mL). The peptide, Lubelisin was added at 3× MIC concentration. Experiments were performed in triplicates, and CFU/mL was calculated at different time points after overnight incubation of inoculated agar plates (MHA) at 37 °C. Kill curves were plotted with CFU/mL against time (h).

### Serial passage for selection of resistance

To evaluate if populations of AMP-resistant bacteria could be selected, cultures were continuously exposed to Lubelisin for a duration of 26 days, as previously described [67]. Briefly, broth microdilution susceptibility testing was performed using a standard doubling-dilution series of Lubelisin concentrations on Day 1. Following incubation of the cultures for 24 h, and determination of the MIC, the well that contained the highest concentration of AMPs permitting growth was diluted 1: 1000 in MHB and used to provide the inoculum for the next MIC assay; this process was repeated daily for 26 days.

### Membrane permeabilisation

Membrane permeabilisation was evaluated using the propidium iodide assay as previously explained [68]. Briefly, logarithmic phase suspension of bacterial cultures was prepared from overnight culture grown in MH broth. Bacteria were pelleted by centrifugation for 5 min at 4000 rpm and resuspended in sterile Phosphate Buffered Saline (PBS) at about 10^9^ bacteria/mL. Propidium iodide was added to the bacterial suspension to a final concentration of 30 μM and allowed to equilibrate for 15 min at 37 °C. This suspension (100 μL) was then transferred into black 96-well plates already containing 100 μL of serially diluted peptides at the desired concentrations. Kinetics of fluorescence variations (excitation at 530 nm / emission at 590 nm) were then recorded using a microplate reader over a 2-h period with incubation at 37 °C. Cetyl trimethylammonium bromide (CTAB) (at 300 μM) served as positive control giving 100% permeabilisation. The permeabilisation effect of Lubelisin was expressed as the percentage relative fluorescence unit (RFU) to the positive control, CTAB.

### Haemolytic activity and therapeutic index measurement

The ability of Lubelisin to lyse erythrocytes was assessed as previously described [17, 69]. Fresh human red blood cells (RBC) (Cambridge Bioscience, UK) and defibrinated sheep erythrocytes (Oxoid Ltd. Hampshire, UK) were washed 3 times with PBS, centrifuged for 5 min at 500 rpm between each wash and resuspended to 4% (v/v) in PBS. This mixture was dispensed in 96-well plates and the peptide, Lubelisin was added to the mixture at a range of concentrations (or at MIC, 2× MIC and 3× MIC values for *S. aureus* strains (i.e., 8, 16 and 24 μg/mL only for haemolytic activity against sheep erythrocytes). The mixtures were incubated at 37 °C for 1 h. Plates were then centrifuged for 5 min at 1000 rpm and the absorbance (OD_450 nm_) of the supernatant was measured using the Synergy™ H4 Hybrid Multi-Mode reader (BioTek Swindon UK) to detect any haemoglobin release. Readings from PBS only wells (0% cell lysis) were used to normalise data from all other wells and 0.1% Triton X-100 served as a positive control (representing 100% cell lysis). The percentage of haemolysis was calculated according to the equation: (*A*_450_ peptide solution − *A*_450_ PBS)/ (*A*_450_ 0.1% Triton X-100 − *A*_450_ PBS) × 100. The therapeutic index (T.I.) was calculated as the ratio of the minimum haemolytic concentration ((MHC)- causing 5% haemolysis to human erythrocytes) to the minimum inhibitory concentration (MIC_GM+_) [46]. The MIC_GM+_ was the minimum inhibitory concentration of the peptide against Gram-positive bacterial strains after the geometric mean was calculated.

### Transmission electron microscopy

Transmission electron microscopy was used to further investigate the mode of action and the effect of Lubelisin [70]. Briefly bacterial cells at mid-log phase were treated with Lubelisin at 3× MIC concentration of the pathogen for 30 min. The treated samples were then washed 3 times at 5000 rpm for 5 min with PBS and fixed with 2.5% glutaraldehyde (Agar Scientific Ltd), in 0.1 M sodium cacodylate buffer pH 7.4 (Agar Scientific Ltd) overnight at 4 °C. The cells were fixed, processed, and adsorbed onto Formvar/carbon-coated copper grids and stained with 2% uranyl acetate (pH 4). Samples were subsequently visualized on a Jeol 1010 transmission electron microscope (JEOL Ltd., Tokyo, Japan) operated at 80 kV and 30,000 × magnifications. Images were recorded with a Kodak MegaPlus camera Model 1.4i, visualized by analySIS 3.1 software and processed on ImageJ.

### Statistical analysis

All comparisons were based on the mean ± standard deviation of the mean (SD). Differences between treatment groups were analysed using two-way analysis of variance (ANOVA) with the Bonferroni post-test method for comparison between groups. Results were considered significant when *P* values were < 0.05.

## Supplementary Information

**Additional file 1.**

## Data Availability

All data generated or analysed during this study are included in this published article [and its supplementary information files]. Sequences are deposited in the Short Read Archive under Bioproject: PRJNA563675 (https://www.ncbi.nlm.nih.gov/bioproject/PRJNA563675); Biosample: SAMN12684929 (https://www.ncbi.nlm.nih.gov/sra/?term=SAMN12684929): HAN4BADXX (https://www.ncbi.nlm.nih.gov/sra/?term=HAN4BADXX) and in the GenBank database under accession numbers MK286889 (https://www.ncbi.nlm.nih.gov/nuccore/MK286889.1/), MK286890 (https://www.ncbi.nlm.nih.gov/nuccore/MK286890) and MK286891 (https://www.ncbi.nlm.nih.gov/nuccore/MK286891).
